# Adaptive Wavelet Coding Applied in a Wireless Control System

**DOI:** 10.3390/s17122901

**Published:** 2017-12-13

**Authors:** Felipe O. S. Gama, Luiz F. Q. Silveira, Andrés O. Salazar

**Affiliations:** Department of Computer Engineering and Automation, Universidade Federal do Rio Grande do Norte, Lagoa Nova, Natal 1524, Brazil; lfelipe@dca.ufrn.br (L.F.Q.S.); andres@dca.ufrn.br (A.O.S.)

**Keywords:** wireless communications, wavelet coding, time diversity, Rayleigh fading, control loop

## Abstract

Wireless control systems can sense, control and act on the information exchanged between the wireless sensor nodes in a control loop. However, the exchanged information becomes susceptible to the degenerative effects produced by the multipath propagation. In order to minimize the destructive effects characteristic of wireless channels, several techniques have been investigated recently. Among them, wavelet coding is a good alternative for wireless communications for its robustness to the effects of multipath and its low computational complexity. This work proposes an adaptive wavelet coding whose parameters of code rate and signal constellation can vary according to the fading level and evaluates the use of this transmission system in a control loop implemented by wireless sensor nodes. The performance of the adaptive system was evaluated in terms of bit error rate (BER) versus $E_{b}/N_{0}$ and spectral efficiency, considering a time-varying channel with flat Rayleigh fading, and in terms of processing overhead on a control system with wireless communication. The results obtained through computational simulations and experimental tests show performance gains obtained by insertion of the adaptive wavelet coding in a control loop with nodes interconnected by wireless link. These results enable the use of this technique in a wireless link control loop.

## 1. Introduction

The traditional communication architectures employed in industrial control and manufacturing systems are generally based on wired systems. According to [1], this type of architecture is generally centralized and, thus, does not provide some desired requirements for modern industrial systems such as: modularity, decentralized control, ease of diagnostics and low cost. An alternative to overcome the difficulties imposed by wired networks is the use of wireless sensors in industrial plants [2]. Flexibility, low cost and scalability are some of the advantages of wireless sensor networks.

However, the use of wireless sensors in industrial environments should ensure low demand for memory and processing resources, low bit error rate, low power consumption, dynamic topology and security [3,4].

Wireless sensors use a communication susceptible to distortions caused by flat fading, produced by multiple propagation paths of the signal, and by shading, as a function of the obstacles present. In an industrial environment in addition to multiple paths and obstacles, there are other sources of noise, such as welding equipment, microwave ovens and wireless communication instruments. Therefore, there is a high level of fading on a large and small scale, according to [5]. Thereby, when transmitted in industrial environments, the signals are subject to distortions and errors caused by the propagation channel, which can cause problems in the controlled and/or monitored equipment. These communication problems are more critical in industry than in telecommunications systems designed for voice transmission.

The use of wireless sensors in a control loop has other challenges to overcome such as packet loss, packet error, routing optimization, topology, latency and jitter, among others. There are several protocols and techniques in the literature that address these challenges, some of which are analyzed by [6,7,8]. The packet loss can be addressed using control techniques, such as the use of a model-based controller, as analyzed by [9].

Another approach used to combat the degenerative effects present in the wireless communications scenarios, and applied in control systems, is the use of filtering, diversity and coding techniques, some of which are analyzed by [10,11,12].

Diversity techniques consist of generating redundancy (replicas) of the transmitted signal at the receiver. They are then transmitted on independent channels, being affected differently in an uncorrelated way by the channel [13]. Mitigation of transmission errors favors the control system, although it can lead to a reduction in the system spectral efficiency.

However, wavelet coding can be used to generate time diversity without compromising spectral efficiency [14]. It was initially proposed by Tzannes et al. in [15] as an alternative channel coding technique to minimize fading effects, based on the orthogonality properties that exist between the rows of the wavelet coefficient matrix (WCM). It is also important to note that wavelets are also used in source coding techniques or even in systems with joint source-channel coding [16,17,18]. However, this work proposes the use of wavelets in an unusual way. Specifically, we use filters based on wavelet coefficients as channel encoders in order to increase the robustness of the communication system in relation to the degenerative effects present in the wireless communication.

In the encoding process considered in this work, each data bit sent to the wavelet encoder is successfully multiplied by the coefficients of a given row in the WCM, thus disseminating information in multiple coded symbols. The resulting encoded symbols are not equiprobable and present multiple levels, being transmitted in different time intervals. Due to the WCM orthogonality properties, the spread information of each bit may be collected in the decoder by proper correlation with the WCM rows used for encoding. This mechanism of spreading the information in time before transmission and gathering it in the receiver potentially increases the communication system robustness to the combination of time-varying fading and localized noise effects.

Wavelet coding was thoroughly evaluated in previous works, which include its property of error correction [15,19,20,21]. The obtained results have shown that such a technique is quite efficient, which makes it suitable for applications in communication systems. Preliminary results evaluating the effects of the addition of wavelet coding on a control system were evaluated in [22]. However, the evaluation of the impacts of this coding technique embedded in control systems has not been presented so far in detail. This paper proposes the design of a control system with adaptive wavelet coding and an evaluation of its performance over flat Rayleigh fading channels. The increase of the robustness in the wireless communication provided by the wavelet coding allows the transmission of auxiliary data, which can be necessary in control system scenarios [23].

The rest of this paper is organized as follows. Section 2 presents the coding and decoding algorithms with matrices of wavelet coefficients. Section 3 presents the control system with adaptive wavelet coding proposed in this paper. The results and discussions are presented in Section 4, and the conclusion are drawn in Section 5.

## 2. Fundamentals of Wavelet Coding

In this technique, a flat (a matrix is called flat if all of its entries have the same absolute value) integer WCM is used to encode information bits. A flat integer matrix $A=(a_{k}^{j})$, where $a_{k}^{j}\in \left\{-1,\;+1\right\}$, of dimension m×mg is said to be a wavelet coefficient matrix of rank m≥2 and genus *g* if it satisfies the modified wavelet scaling conditions given by [15,24]:

(1) $$\sum_{k=0}^{mg-1}a_{k}^{j}=m\sqrt{g}\delta_{0,j},\;\;0\leq j\leq m-1$$

(2) $$\begin{array}{cc} \sum_{k}a_{\left[k+ml\right]}^{j}a_{\left[k+ml^{\prime }\right]}^{j^{\prime }}=mg\delta_{j,j^{\prime }}\delta_{l,l^{\prime }}, & 0\leq j,j^{\prime }\leq m-1\\ & 0\leq l,l^{\prime }\leq g-1 \end{array}$$

where $\delta_{j,j^{\prime }}$ is the Kronecker delta and the notation [k+ml] stands for the operation k+ml modulus mg.

The fundamental property of a WCM for channel coding purposes is given by Equation (2). It states that the rows of a given WCM with order *m* are mutually orthogonal at shifts of length lm, where 0≤l≤g−1. Besides, each row is also orthogonal to a copy of itself shifted by lm, where 0<l≤g−1.

### 2.1. Wavelet Encoding Algorithm with Variable Code Rate

So far, approaches employing wavelet coding in transceivers use coding rates equal to one information bit per channel symbol. In this paper, we propose a novel adaptive wavelet-coded system whose coding rate changes according to the channel state.

In the proposed approach, the adaptive coding rate can be easily obtained by adjusting the displacement between the rows of a wavelet coding matrix $C_{WCM}$ per m/k, for integer 1/k, where *k* is the code rate, without losing the orthogonality properties of the matrix, fundamental for this coding technique, as discussed as follows.

**Figure 1:**
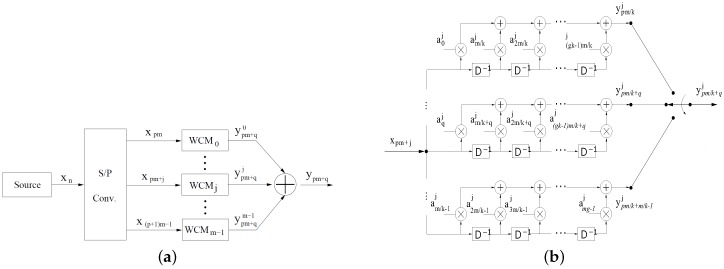
Encoder overview (**a**) and detailed view of $MCW_{j}$ blocks of the wavelet encoder (**b**).

At each instant n=pm/k+q, where in p∈{0,1,2,3,4,⋯} and q∈{0,1,⋯,m/k−1}, there will be m/k wavelet sub-symbols $y_{pm+q}^{j},0\leq j\leq m/k-1$, generated simultaneously by the register *q*-th of each of the *m* banks $WCM_{j}$ and available at the output of wavelet encoder. In Figure 1b, it can be observed that the wavelet sub-symbol $y_{(pm/k)+q}^{j}$, by register *q*-th of bank $WCM_{j}$, is given by:

(3) $$\begin{array}{c} y_{(pm/k)+q}^{j}=\sum_{l=0}^{gk-1}a_{(lm/k)+q}^{j}x_{(p-l)m+j}. \end{array}$$

As there are mg/k elements in each bank of shift registers, as shown in Figure 1b, each input bit can affect at most mg/k wavelet sub-symbols. Finally, the m/k wavelet sub-symbols with the same time index n=(pm/k)+q are then added, and the resulting wavelet symbol is given by:

(4) $$\begin{array}{c} y_{(pm/k)+q}=\sum_{j=0}^{m-1}\sum_{l=0}^{gk-1}a_{lm/k+q}^{j}x_{(p-l)m+j}. \end{array}$$

The proposed wavelet coding process can also be written as the matrix product, as shown in the equation below:

(5) $$\begin{array}{c} \bar{y}=\bar{x}\cdot C_{MCW}. \end{array}$$

Table 1 shows the wavelet symbols in relation to time, wherein it presents in detail Equation (5).

As an illustration, Table 2 and Table 3 show the symbols generated by the wavelet encoder based on the WCM2×8 for k=1 and k=0.5, respectively.

### 2.2. Wavelet Decoding Algorithm

**Figure 2:**
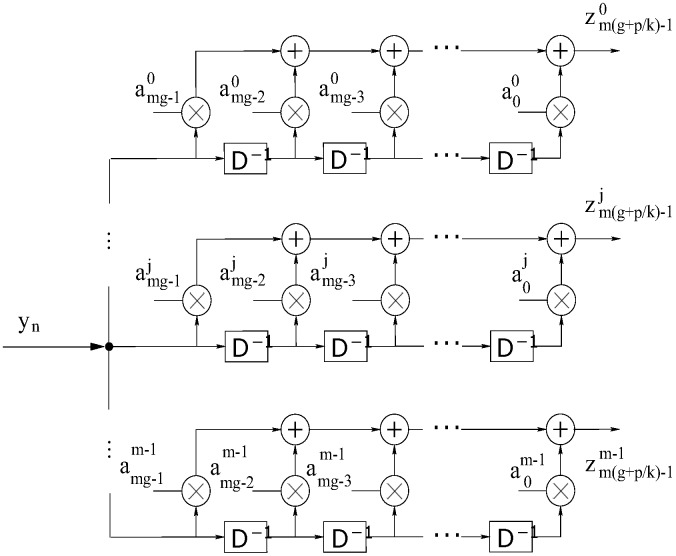
General structure of the wavelet decoder.

At reception, the sequence of information bits $x_{n}$ can be retrieved from the symbol sequence $y_{n}$ received using a bank with *m* correlators of mg length, matched to the *m* lines of WCM used in wavelet coding. Figure 2 shows the general structure of a decoder based on Equation (6). Assuming the absence of noise, the correlator output $z^{j}$, j∈{0,1,…,m−1}, matches the $a^{j}$ line of WCM, at the instant of time i=m(g+p/k)−1, wherein p∈Z is given by:

(6) $$\begin{array}{cc} z_{i}^{j} & =\sum_{k=0}^{mg-1}a_{mg-1}^{j}y_{i-k}\\ & =\sum_{k=0}^{mg-1}\sum_{j^{\prime }=0}^{m-1}\sum_{l=0}^{g-1}a_{k}^{j}\left(a_{k-lm}^{j^{\prime }}x_{j^{\prime }+lm+i-\left(mg-1\right)}\right)\\ & =x_{j+i-(mg-1)}\sum_{k=0}^{mg-1}a_{k}^{j}a_{k}^{j}\\ & =mgx_{j+i-(mg-1)} \end{array}$$

Due to the interference caused by the communication channel on the transmitted wavelet symbols, the estimates of the information bits are defined by ${\hat{x}}_{j+i-(mg-1)}=\mathrm{sgn}\left(z_{i}^{j}\right)$.

Finally, it is important to note that the difference between the unitary code rate algorithm and the variable code rate algorithm is only the frequency at which the decoder output is observed, since with the variable rate, obtaining estimates of Mbits transmitted occurs every m/k instants of received wavelets symbols.

## 3. System Description

**Figure 3:**
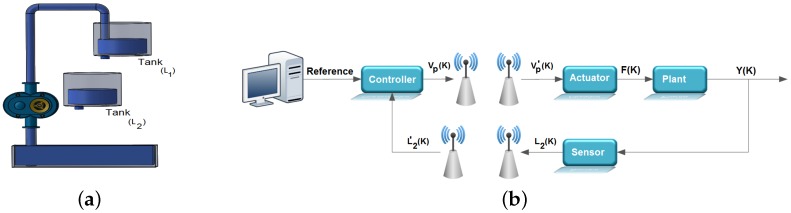
(**a**) System coupled tanks; (**b**) diagram of the control system adopted in this work.

Figure 3b shows an overview of the control loop considered in the work, it being evident in this figure that the feedback and control signals are transmitted through wireless links. This control loop is adopted for the computational simulations and for the experimental tests carried out in this work. To carry out these experimental tests, a test bench was developed in which the wavelet coding is embedded in an ATMEGA 328p microcontroller associated with the Arduino UNO development kit. The experimental tests were carried out with the sole purpose of observing the processing time associated with the wavelet coding calculations and thus verifying if the addition of wavelet coding in the control loop could affect the performance of the system controller. In these experimental tests, the radio frequency (RF) module NRF24L01, from Nordic, was used for the transmission of RF signals needed to control the system under consideration.

In the context of this work, the evaluated system consists of a reservoir, a pump and two vertical tanks. The two tanks contain a hole in their base, which allows the flow of water, the upper tank receiving water pumped from the reservoir. Thus, the upper tank feeds the bottom tank through the hole in the base, and the lower tank closes the cycle with water returning to the reservoir by its bore. Despite other possibilities, the configuration adopted was a SISO (single-input-single-output) system, according to Figure 3a.

### 3.1. Control Loop Developed for Experimental Testing

**Figure 4:**
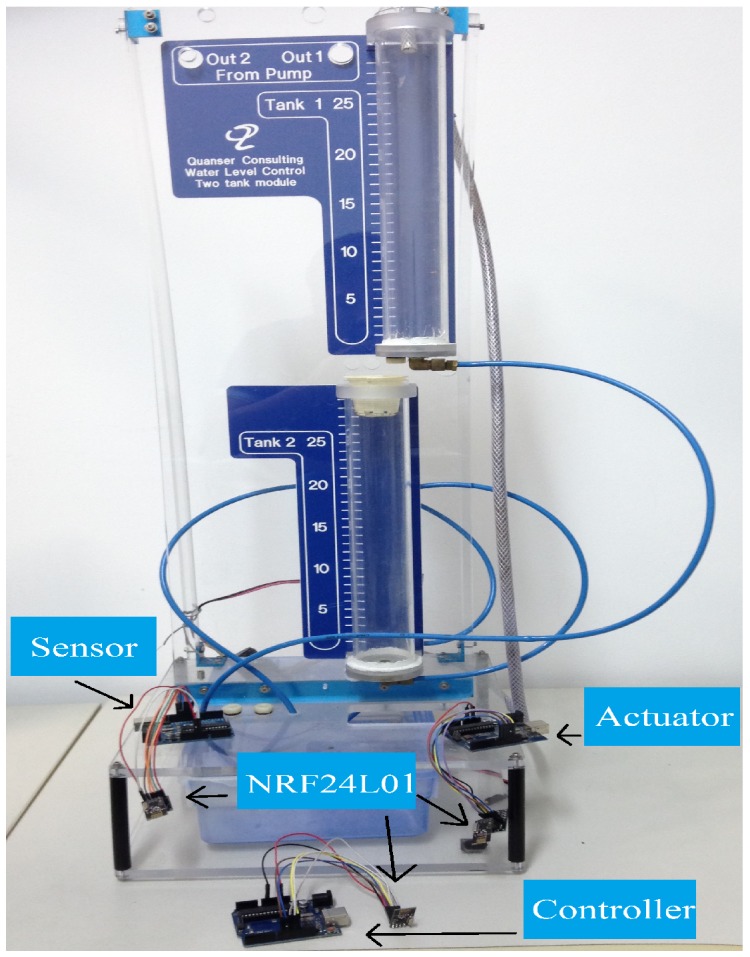
Benchtop developed for experimental tests.

**Figure 5:**
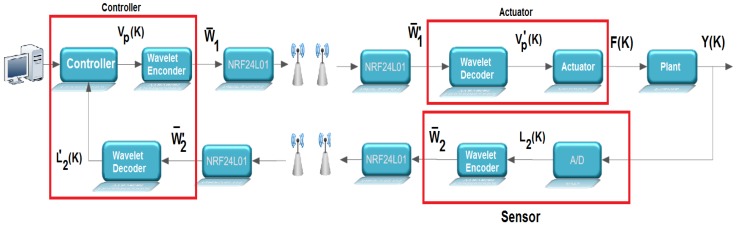
Detailed view of the structure of the control loop developed to perform the experimental tests.

The illustration in Figure 4 gives an overview of the test benchtop design. It is basically composed of three microcontrollers, represented by: sensor, controller and actuator, in accordance with the system shown in Figure 5.

When the system is running, the sensing element receives a voltage signal referring to the level of the tank to be controlled (Y(k)) from the plant by wired communication. The analog-to-digital converter (A/D) with a resolution of 10 bits, present in the sensor and configured with a conversion period of $t_{s}$, accomplishes the conversion of that signal, obtaining $L_{2}\left(k\right)$. After the conversion, the sensor itself performs the wavelet encoding of the $L_{2}\left(k\right)$ signal. Each conversion is carried out for a period $t_{s}$. The encoded symbols are transmitted through the radio frequency module NRF24L01 to the controller, which decodes these symbols and executes a PID control routine with reference to the value of the setpoint. The control signal obtained by the PID algorithm ($v_{p}$) is coded, then these symbols are transmitted by the radio frequency module to the actuator. The actuator decodes the received symbols by wireless communication and produces a PWM signal to be injected into the circuit.

This paper adopts a wireless communication scenario, in which the nodes are in an outdoor environment, with the Doppler effect; for example, this scenario is the control of the turbine used in wind power systems [10,25].

### 3.2. Implemented Control Loop for Performing Computational Simulations

**Figure 6:**
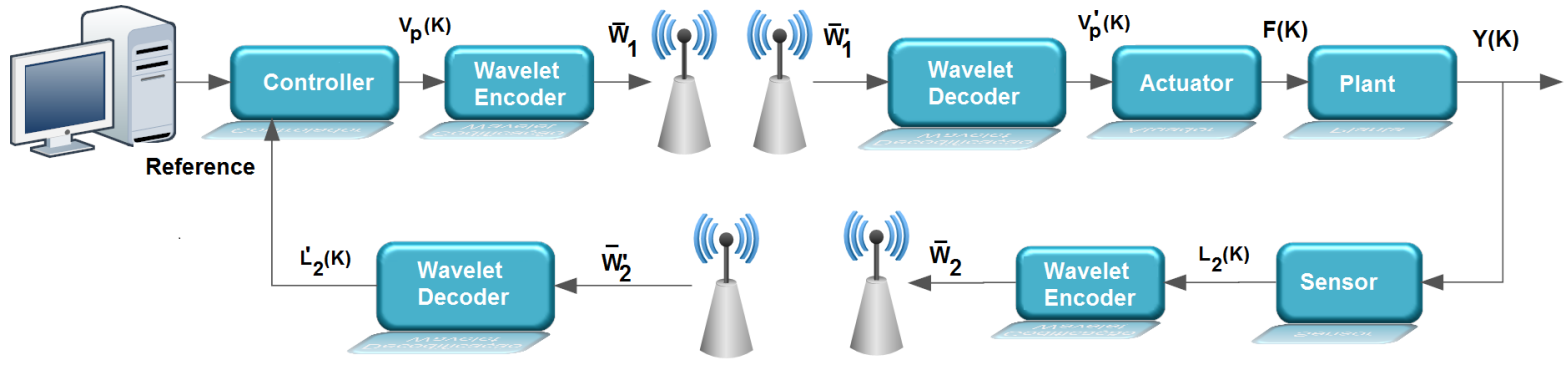
Control loop structure implemented for performing computer simulations.

In this work, the computational simulations analyze the impact of the degenerative effects caused by the wireless communications in a control loop. In the block diagram of Figure 6, the sensor receives from the plant, by wired communication, a signal related to the level of the tank to be controlled ($L_{2}$), then performs the wavelet coding of that signal. The coded symbols are then transmitted to the controller via a channel with Rayleigh fading and with Doppler shift. Each sample of the signal to be controlled is taken in a period $t_{s}$. The controller decodes these symbols and performs a PID control routine based on the value of the setpoint set on the computer. The control signal ($v_{p}$) obtained by the PID algorithm is coded, and the generated symbols are transmitted to the actuator by a channel with flat Rayleigh fading and with the Doppler effect. The actuator decodes the received symbols via wireless communication and injects that signal into the plant. It is important to note that the signal $v_{p}$ is limited to an operating region between 0 V and 5 V.

### 3.3. Implementation of an Adaptive Wavelet Coding

Adaptive coding and modulation techniques seek to adapt the transmission parameters of a system to the conditions of time-varying channels in order to ensure a more robust and spectrally-efficient transmission [26,27]. The fundamental parameters to be adapted include coding and modulation, but other parameters can be adjusted, such as power levels. The basic premise of these techniques is to estimate the channel at the receiver and send that estimate back to the transmitter so that the transmission scheme can be adapted according to the channel characteristics.

Adaptive coding and modulation for transmitting fading over the channel may lead to an increase of the average code rate, reduction of the required transmission power or reduction of the bit error probability, taking advantage of the favorable channel conditions [28].

This paper proposes the use of adaptive wavelet coding and modulation applied in a control loop over a wireless link to send the feedback and control signals. Thus, when the channel is in a good state, the code rate will increase, enabling the dispatch of auxiliary data at this time without compromising the main data flow of the control system. In addition, the use of an adaptive encoder enables a low processing overhead and consequently a good energy efficiency. This parameter is very important in a wireless sensor network.

The adaptive coding is simulated considering that the transmitter and receiver have perfect information about the current channel state. Taking this information into account, the system can choose the wavelet coding and modulation scheme used in the transmission.

**Figure 7:**
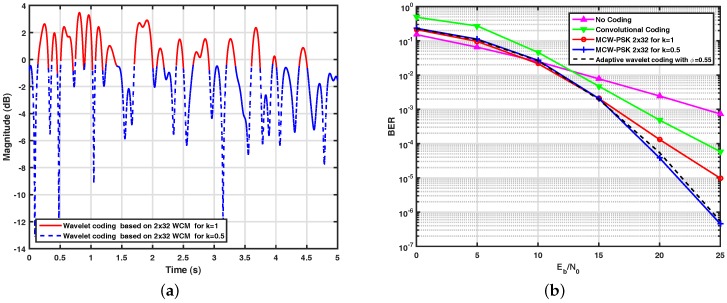
(**a**) Illustration of the behavior of the implemented wavelet adaptive encoder; (**b**) system performance with adaptive wavelet coding.

The adaptive wavelet encoder used in this work was designed with the assistance of the Markov chain. In the proposed architecture, the input symbols are encoded by the 2×32 WCM with k=1 when the path gain values of the fading channel are greater than 1.5, and they are encoded by the 2×32 WCM with k=0.5, when the path gain values are less than or equal to 1.5. Figure 7a illustrates the simulated adaptive wavelet encoder. In this figure, it is possible to verify each encoder operating region according to the proposed architecture. The spectral efficiency estimate of this encoder is equal to 0.55 bits/s/Hz.

When compared to conventional channel coding techniques, in a similar environment, the strategy for wavelet coding presents satisfactory gains. Figure 7b shows performance curves obtained through computational simulations of adaptive wavelet coding based on the 2×32 WCM, previously described, and the wavelet coding systems based on 2×32 WCM for k=1 and k=0.5, the latter with spectral efficiency equal to 1 bits/s/Hz and 0.5 bits/s/Hz, respectively. For the purpose of comparison, we also present in this figure the performance of an uncoded BPSK system and a system with convolutional coding with the polynomial generator (151, 144), a restriction length of 7 and a code rate of 0.5, associated with a QPSK modulation scheme, and a Viterbi hard receiver. From this figure, it can be observed that the performance gain of the proposed adaptive system, in terms of BER versus $E_{b}/N_{0}$, can reach two decades when we compare its performance curve with the curve of the system based on the convolutional encoder, in $E_{b}/N_{0}=25$ dB.

As can be seen, the performance of the proposed adaptive wavelet coding has similar performance to the non-adaptive wavelet-coded system based on the 2×32 WCM with k=0.5, but with a spectral efficiency equal to 0.55 bit/s/Hz, which corresponds to a spectral efficiency gain of approximately 10% in relation to that reference system. In particular, it can be observed that the proposed adaptive wavelet coding allows for doubling the transmission rate when the channel is in a good state without degrading the system’s average performance in terms of bit error rate, which allows the sensors to send auxiliary data; for example, the status of the battery without compromising the basic data flow for the control loop.

## 4. Obtained Results

This section presents the results obtained through experimental tests and computational simulations of the models described previously for the considered control system. In order to analyze the impact of the processing time of the wavelet coding in a control loop that operates on the wireless link, the processing time of the wavelet coding and the performance of the controller with and without the coding technique were evaluated in the absence of channel errors. The purpose is to investigate if the insertion of the wavelet coding in a control loop generates some significant delay, which could affect the control of the system.

Computational simulations were also carried out to evaluate the control system performance over the Rayleigh fading channel with the normalized Doppler in $f_{D}T_{s}=0.002$ for different $E_{b}/N_{o}$ values. During the simulations, $10^{3}$ data packets were evaluated for each setpoint value.

### 4.1. Analysis of the Impact of Wavelet Coding Processing Time on a Control Loop

In order to verify the impact of the use of wavelet coding in a control system, two communication scenarios were evaluated. In the first one, the control loop with wired communication was simulated computationally, in order to tune the controller embedded in the microcontroller. In the second one, experimental tests involving the analysis of a control system using the wavelet coding were carried out in order to investigate the impact of the coding processing time on the controller performance. In both scenarios, the absence of errors caused by the communication channel was admitted.

**Figure 8:**
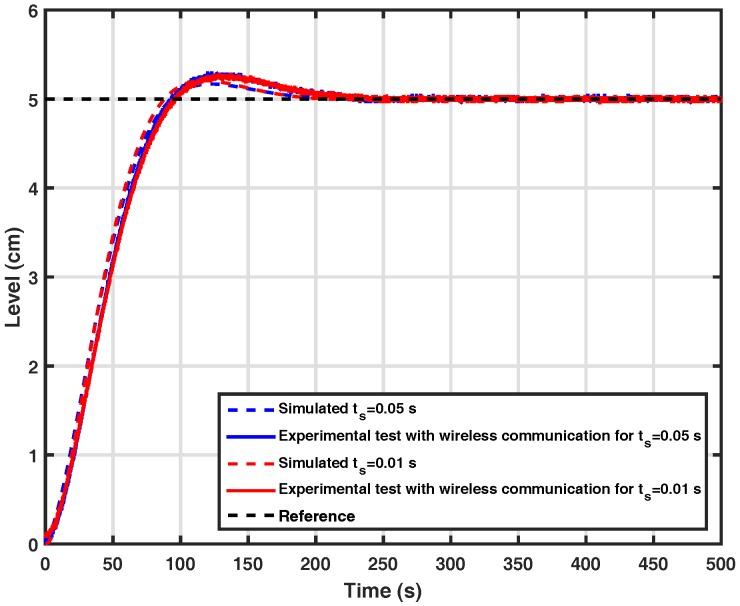
System response for both scenarios.

Figure 8 shows the performance curves of the control system obtained for the two scenarios. It can be observed in this figure that the addition of the wavelet coding does not generate delay in this control system, therefore leading the system to an instability condition. For a better analysis of the two results, it is important to mention that the two scenarios adopt the same configuration: a plant of second order, in which the pump transfers the liquid to Tank 1. The orifice at its base allows this liquid to flow into the Tank 2, whose level must be controlled, and the controller used is a PID, with $K_{p}=1.5$, $k_{i}=0.04$ and $k_{d}=0.05$. Tests were carried out with different periods of sampling with the objective of evidencing the impact of processing time on the control system.

In addition, the processing time of the encoder and decoder embedded in the ATMEGA 328P microcontroller with a clock of 16 MHz was also measured. The obtained values were around 450μs and 180μs, respectively. From Figure 8, it can be observed that the time scale of the wavelet coding in μs does not generate delay in a control system and therefore does not harm the controllability of the system. The encoder and decoder processing times were measured with timers present on the microcontroller used.

### 4.2. Control System with Adaptive Wavelet Coding over the Channel Subject to Rayleigh Fading

For all results presented in this subsection, the variable to be controlled is the level of the second tank ($L_{2}$), and the controller gains are $K_{p}=1.5$, $k_{i}=0.04$ and $k_{d}=0.05$. The signals received by the controller and actuator are denoted by $L_{2}^{\prime }$ and $v_{p}^{\prime }$, respectively, and the signals sent by the sensor and controller are denoted by $L_{2}$ and $v_{p}$, respectively.

**Figure 9:**
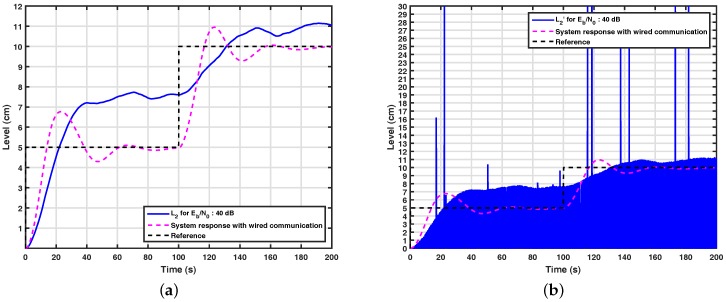
(**a**) System response without wavelet coding for various signal-to-noise ratios; (**b**) signal received by the controller on a system without wavelet coding.

Figure 9a shows the system response of a control loop over a wireless link, but without wavelet coding for a $E_{b}/N_{0}$ equal to 40 dB. This wireless system uses BPSK modulation and has spectral efficiency equal to 1 bit/s/Hz. From this figure, it can be seen that even at high signal-to-noise ratios, the system is not controllable, since the degenerative effects present in the wireless channel cause an inversion on the bits received by the controller and actuator, distorting the values of those messages. The change of the values received by the controller can be seen in Figure 9b, wherein these messages are distorted in such a way that $L_{2}$ is degenerated.

**Figure 10:**
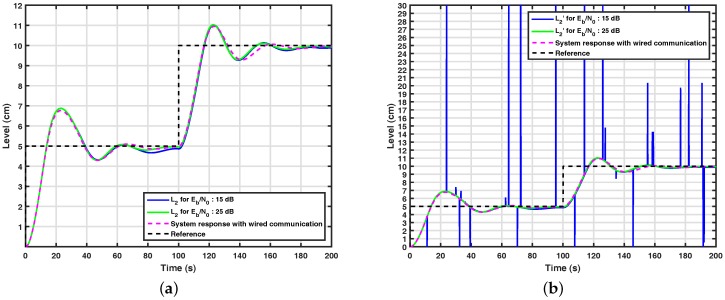
(**a**) System response with based wavelet coding based on 2×32 WCM for k=1; (**b**) signal received by the controller in a system with wavelet coding based on 2×32 WCM for k=1.

**Figure 11:**
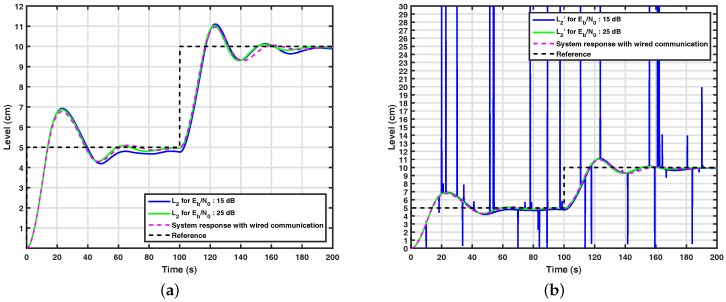
(**a**) System response with wavelet coding based on 2×32 WCM for k=0.5; (**b**) signal received by the controller in a system with wavelet coding based on 2×32 WCM for k=0.5.

Figure 10a and Figure 11a show the results obtained through computational simulations for the system with the wavelet coding based on 2×32 WCM for k=1 and k=0.5 in the control loop. It can be seen from this figure that the control system with wavelet coding appears to be controllable from $E_{b}/N_{0}=15$ dB over a channel with the normalized Doppler in $f_{D}T_{s}=0.002$, since it has similar performance to the system simulated over wired communication, also shown in the figure. From Figure 9a, Figure 10a and Figure 11a, it can be noticed that the control system with wavelet coding behaves satisfactorily in the considered communication scenarios, but when the control system does not have a channel coding technique equivalent to wavelet coding, the control loop is not controllable, even for high signal-to-noise ratios, which reinforces the importance of wavelet coding in these scenarios. It is also important to note that with the addition of wavelet coding, fewer errors occur, as shown in Figure 10b and Figure 11b.

**Figure 12:**
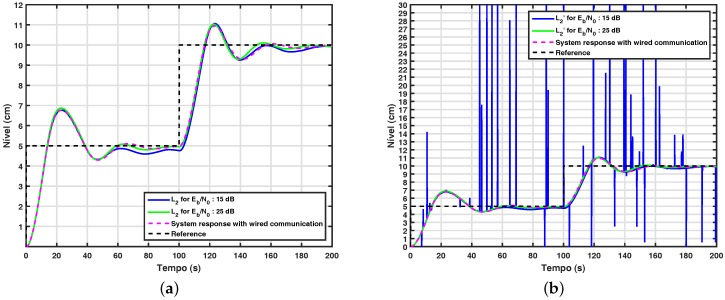
(**a**) System response with adaptive wavelet coding; (**b**) signal received by the controller in a system with adaptive wavelet coding.

Figure 12a shows the behavior of the control system with the adaptive wavelet coding proposed in this work. In order to evaluate the performance of the proposed control system, a control loop with wired communication between the nodes is also simulated and used as a reference system. From this figure, it can be observed that the system behaves properly when $E_{b}/N_{0}\geq 15$ dB, since in this case, it has performance similar to the reference system. It is important to note that most wireless systems only operate properly in channels with signal-to-noise equal to 20 dB, indicating that the results obtained from these experiments are satisfactory. Finally, it is important to note that the adaptive wavelet coding has a spectral efficiency of 0.55 and a BER less than $10^{-6}$ in $E_{b}/N_{o}$, equal to 25 dB, so the adaptive wavelet coding proposed in this work meets the requirements of flexibility and robustness required in a control loop.

## 5. Conclusions

This paper proposes the design and evaluation of control systems with adaptive wavelet coding over flat Rayleigh channels. The results indicate that the proposed coding technique has a good performance in terms of BER and that the processing time of this technique does not generate a delay, in such a way that it sends the control loop to a point of instability.

Besides, the use of wavelet coding allowed a good performance of the control system over wireless channels, even in the presence of transmission errors caused by the channel in scenarios of low $E_{b}/N_{o}$ values. The function of the wavelet coding on the control system performance is especially evident when analyzing the curves of the signal received by the controller in a scenario without the channel encoder. In this case, the system becomes uncontrollable, even at high $E_{b}/N_{0}$ values.

## Figures and Tables

**Table 1:** Relationship in time between incoming bits and wavelet symbols.

n	0	1	⋯	m/k	⋯	2g−1	2g	⋯
$x_{0}\cdot$	$(a_{0}^{0}$	$a_{1}^{0}$	⋯	$a_{m/k}^{0}$	⋯	$a_{2g-1}^{0})$		
$x_{1}\cdot$	$(a_{0}^{1}$	$a_{1}^{1}$	⋯	$a_{m/k}^{1}$	⋯	$a_{2g-1}^{1})$		
$x_{2}\cdot$				$(a_{0}^{0}$	$a_{1}^{0}$	$a_{2}^{0}$	⋯	$a_{2g-1}^{0})$
$x_{3}\cdot$				$(a_{0}^{1}$	$a_{1}^{1}$	$a_{2}^{1}$	⋯	$a_{2g-1}^{1})$
⋮						⋮	⋮	⋱
	$y_{0}$	$y_{1}$	⋯	$y_{m/k}$	⋯	$y_{2g-1}$		

**Table 2:** Wavelet symbols produced by the encoder with 2×8 wavelet coefficient matrix (WCM) for k=1.

${\mathrm{nT}}_{s}$	$y_{n}$
0	$a_{0}^{0}x_{0}+a_{0}^{1}x_{1}$
1	$a_{1}^{0}x_{0}+a_{1}^{1}x_{1}$
2	$a_{2}^{0}x_{0}+a_{2}^{1}x_{1}+a_{0}^{0}x_{2}+a_{0}^{1}x_{3}$
3	$a_{3}^{0}x_{0}+a_{3}^{1}x_{1}+a_{1}^{0}x_{2}+a_{1}^{1}x_{3}$
4	$a_{4}^{0}x_{0}+a_{4}^{1}x_{1}+a_{2}^{0}x_{2}+a_{2}^{1}x_{3}+a_{0}^{0}x_{4}+a_{0}^{1}x_{5}$
5	$a_{5}^{0}x_{0}+a_{5}^{1}x_{1}+a_{3}^{0}x_{2}+a_{3}^{1}x_{3}+a_{1}^{0}x_{4}+a_{1}^{1}x_{5}$
6	$a_{6}^{0}x_{0}+a_{6}^{1}x_{1}+a_{4}^{0}x_{2}+a_{4}^{1}x_{3}+a_{2}^{0}x_{4}+a_{2}^{1}x_{5}+a_{0}^{0}x_{6}+a_{0}^{1}x_{7}$
7	$a_{7}^{0}x_{0}+a_{7}^{1}x_{1}+a_{5}^{0}x_{2}+a_{5}^{1}x_{3}+a_{3}^{0}x_{4}+a_{3}^{1}x_{5}+a_{1}^{0}x_{6}+a_{1}^{1}x_{7}$
⋮	⋯

**Table 3:** Wavelet symbols produced by the encoder with 2×8 WCM for k=0.5.

${\mathrm{nT}}_{s}$	$y_{n}$
0	$a_{0}^{0}x_{0}+a_{0}^{1}x_{1}$
1	$a_{1}^{0}x_{0}+a_{1}^{1}x_{1}$
2	$a_{2}^{0}x_{0}+a_{2}^{1}x_{1}$
3	$a_{3}^{0}x_{0}+a_{3}^{1}x_{1}$
4	$a_{4}^{0}x_{0}+a_{4}^{1}x_{1}+a_{0}^{0}x_{2}+a_{0}^{1}x_{3}$
5	$a_{5}^{0}x_{0}+a_{5}^{1}x_{1}+a_{1}^{0}x_{2}+a_{1}^{1}x_{3}$
6	$a_{6}^{0}x_{0}+a_{6}^{1}x_{1}+a_{2}^{0}x_{2}+a_{2}^{1}x_{3}$
7	$a_{7}^{0}x_{0}+a_{7}^{1}x_{1}+a_{3}^{0}x_{2}+a_{3}^{1}x_{3}$
8	$a_{4}^{0}x_{2}+a_{4}^{1}x_{3}+a_{0}^{0}x_{4}+a_{0}^{1}x_{5}$
9	$a_{5}^{0}x_{2}+a_{5}^{1}x_{3}+a_{1}^{0}x_{4}+a_{1}^{1}x_{5}$
⋮	⋯
